# The association between parity and hypertension: a cross-sectional, community-based study

**DOI:** 10.3389/fcvm.2023.1247244

**Published:** 2023-10-23

**Authors:** Imad R. Musa, Osman E. Osman, Ishag Adam

**Affiliations:** ^1^ Department of Medicine, Royal Commission Hospital at AL Jubail Industrial City, Al Jubail, Saudi Arabia; ^2^ Faculty of Medicine, Alneelain University, Khartoum, Sudan; ^3^ Department of Obstetrics and Gynecology, Unaizah College of Medicine and Medical Sciences, Qassim University, Unaizah, Saudi Arabia

**Keywords:** parity, age, hypertension, associated factor, Sudan, body mass index

## Abstract

**Background:**

The available data on the association between parity and hypertension are inconclusive. This study was conducted to investigate the prevalence of hypertension and its association with parity among adult Sudanese women.

**Methods:**

A multi-stage sampling survey was conducted in four villages in the River Nile State in Sudan between July and September 2022. The World Health Organization's three-level stepwise questionnaire was used to gather the participants' sociodemographic characteristics (age, sex, marital status, parity, educational level, occupation, obstetric history, family history of hypertension, weight and height). Regression analyses were performed.

**Results:**

A total of 408 women were recruited. The median [measured in terms of interquartile range (IQR)] age was 45.0 years (33.0–55.7 years). A linear regression analysis revealed a significant association between parity and diastolic blood pressure (coefficient, 0.60; *P* = 0.011). The prevalence of hypertension (55.9%) increased with parity and ranged from 43.7% to 74.9%. In the multivariate analyses, increasing age (adjusted odds ratio [AOR], 1.03; 95% confidence interval [CI], 1.02–1.05), increasing parity (AOR, 1.09; 95% CI, 1.01–1.19), family history of hypertension (AOR, 1.79; 95% CI, 1.15–2.77), and increasing body mass index (AOR, 1.09; 95% CI, 1.05–1.13) were associated with hypertension. In women of ages ≥ 50 years, increasing parity was significantly associated with hypertension (AOR, 1.15; 95% CI, 1.2–1.29). Para > 5 (AOR, 2.73; 95% CI, 1.11–6.73) was associated with hypertension.

**Conclusion:**

A high prevalence of hypertension was found among Sudanese women, and that parity at 5 or more is linked to hypertension.

## 1. Introduction

Hypertension is one of the major non-communicable diseases (1). Around one-third (31.1%) of the global adult population (1.39 billion people) have hypertension (1, 2). Low- and middle-income countries (31.5%) and African countries (46.0%) (3) have higher prevalence rates of hypertension than high-income countries (28.5%) (1, 2). Sociodemographic, environmental, behavioural factors, high sodium intake, low potassium intake, obesity, alcohol consumption, smoking, lack of physical activity, and nutrition are the identified risk factors for developing hypertension (1, 2). Hypertension is the leading preventable risk factor of cardiovascular diseases and all-cause mortality across the globe (4). Several factors such as lack of awareness of health status, delayed diagnosis, poorly controlled hypertension, and a weak health system expose patients with hypertension in Africa to the highest risk of stroke and heart and renal diseases (3). Ethnicity and race can influence the management of hypertension and its related complications (5). In the global initiatives of the International Society of Hypertension for the screening and management of hypertension, early diagnosis and treatment of hypertension are recommended (6, 7).

The effect of parity on blood pressure levels or hypertension has been reported in several studies (8–13). This may be explained by increased blood volume, increased heart rate, altered myocardial contractility, and reduced afterload and preload, which lead to expanded cardiac output during pregnancy (14). While some studies have shown that parity is associated with an increased risk of developing hypertension (11, 12, 15), others have reported no such association (16). Most of these studies were conducted outside of sub-Saharan African countries. A higher prevalence of parity among women in sub-Saharan Africa was recently reported (17). In studies on the global epidemiology of hypertension, Sudan was identified as one of the countries with a hypertension prevalence rate >34% (1, 2). This is consistent with the findings of some recently published studies on the prevalence of hypertension among the general Sudanese population (35.2%‒41.0%) (18, 19). A higher prevalence of hypertension among females was reported in Eastern Sudan (41.0%) (19). Sudanese women have high parity, with most of them having five deliveries (grand multiparity) at a younger age, before 35 years old (20). Given the importance of the two clinical entities, their potential coexistence, and the meagre published clinical data on this issue in Sudan and in the region, the present study aimed to investigate the prevalence of hypertension among Sudanese women and the influence of parity, especially high parity, and other factors on the development of hypertension among Sudanese women.

## 2. Materials and methods

### 2.1. Study design

This study was conducted in accordance with the principles stipulated in the Declaration of Helsinki. Ethical approval was obtained from the health authority of Almatamah, Sudan (reference No. 03/2021). Signed written informed consent forms were collected from all participants. A multi-stage sampling study was conducted in the River Nile State, northern Sudan, between July and September 2022. River Nile State is one of the 18 states of Sudan and has a total population of 1,120,441 (21). Almatamah is one of the seven localities (the smallest administrative unit in Sudan) in River Nile State and was initially selected by simple random sampling. Adult women in the households of four villages (Hajer Alteer, Athawra Kabota, Alkoumer, and Wadi Alshohda) were selected randomly from the Almatamah locality on the basis of the population size of all sectors. The Strengthening the Reporting of Observational Studies in Epidemiology (STROBE) standard checklists were followed (22). Only Sudanese women (>18 years of age) from the selected households who agreed to participate in the study were selected. Two trained medical officers interviewed the participants during the study period.

### 2.2. Participants

After signing an informed consent form, the participants completed a questionnaire that collected their sociodemographic information, clinical and physical measurements, blood pressures, and weights and heights. Pregnant women; those with known causes of secondary hypertension, renal diseases, medication use (steroid therapy), substance abuse, mental illness, disabilities, or congenital deformities; and those who refused to participate in the study were excluded. The World Health Organization's (WHO) three-level stepwise approach questionnaire was used to collect data (23) on the participants' sociodemographic characteristics, including age; marital status, categorised as married, widow, or divorced; educational level (≤secondary level or >secondary level); and past medical history of hypertension and drug history (steroid therapy). Moreover, a detailed history was obtained regarding the women's menopausal status, history of miscarriage, and live birth/parity. According to the Sudanese tradition, smoking and alcohol consumption are not female habits; hence, we did not include these in the questionnaire to avoid a loss of cooperation among the participants.

An OMRON 3 (with an appropriate cuff size) automated blood measuring device was used to obtain two blood pressure readings after the participant had rested for at least 10 min. The measurement was performed with the participant's arm placed at the level of the heart. The mean of two blood pressure readings (at an interval of 1–2 min) was computed and registered. When the difference between the two readings was significant, that is, >5 mmHg, new measurements were taken until a stable reading was obtained. The method adopted for measuring blood pressure was based on recent recommendations and requirements (24). Women were considered hypertensive on the basis of a reading of ≥140 mmHg for systolic blood pressure and ≥90 mmHg for diastolic blood pressure or both of them. Both criteria were recorded in repeated measurements or reported as having previous hypertension and receiving anti-hypertensive medications (1). The body mass index (BMI) was calculated from the patient's weight and height and grouped according to the WHO's classification for females as follows: underweight (<18.5 kg/m^2^), normal weight (18.5–24.9 kg/m^2^), overweight (25.0–29.9 kg/m^2^), or obese (≥30.0 kg/m^2^) (25).

Parity is defined as the number of times a woman had given birth to a foetus with a gestational age of ≥24 weeks, irrespective of whether the baby was born alive or stillborn (26)*.* Nulliparity is considered as para 0, or no previous delivery, and multipara is defined as a woman who has given birth 2 or more times (26).

#### 2.2.1. Sample size

A sample size of 408 women was calculated using the Open Epi Menu (27), with an assumption of a type I error of 5% and an adequate power of 80% (*β* = 0.2). The estimated sample size (*n* = 408) was calculated on the basis of the assumed hypertension prevalence rate (41.0%) among women. This assumption was based on our previous observations in eastern Sudan (19). Thus, the ratio of women with hypertension to women without hypertension was expected to be 2:3. We assumed that 27.0% of women with hypertension and 15.0% of women without hypertension would have a para ≥5. This assumption was based on our recent work on reproductive health in Sudan (28).

### 2.3. Statistical analysis

The Statistical Package for the Social Sciences (SPSS) for Windows (IBM SPSS v.25) was used to analyse the data. The chi-square test was used to compare the proportions between the women with and those without hypertension. Continuous data were assessed for normality using the Shapiro-Wilk test. A *t*-test and the Mann-Whitney test were used to compare the normally distributed and non-normally distributed data, respectively, between the two groups of women (hypertensive and non-hypertensive). Spearman correlations were performed between continuous variables (age, parity and BMI). Multiple linear regression analysis was conducted for parity with systolic and diastolic blood pressures to assess the risk factors. Logistic regression analyses were performed by entering the dependent (hypertension) and independent variables (age, BMI, educational level, occupation, past medical history of hypertension, and live birth/parity number). Variance inflation factor (<4) and the presence of high correlations (*r* = 0.9) were used to assess the presence of multi-collinearity and there was no multi-collinearity between the independent variables including age, parity and BMI. The independent variables with a univariate *P* value < 0.20 were entered into the model. The adjusted odds ratio (AORs) and 95% confidence intervals (Cis) were calculated, with *P* values < 0.05 considered statistically significant. Backward likelihood ratio adjustments were then performed in the different models.

## 3. Results

Four hundred and eight women were enrolled in this study. Their median [interquartile range (IQR)] age was 45.0 years (33.0‒55.7 years). Their parity ranged from 0 to 10, with a median of 2. A total of 158 women (38.7%) were nulliparous, whereas 95 (23.3%), 73 (17.9%), and 82 (20.1%) had para 1 to 3, 4 or 5, and more than 5, respectively. Of the 408 women, 276 (67.6%) had an educational level ≥ secondary education, and 223 (54.7%) were housewives. Age increased with parity, and women who had para >5 had the highest median (IQR) age [53.0 years (42.0‒60.0), years]. Of 408 enrolled women, 131(32.1%), 39 (9.6%), 123(30.1%) and 115 (28.2%) were normal weight, underweight, overweight and obese, respectively. The women who had para 4 or 5 had the highest BMI [27.6 kg/m^2^ (23.7‒32.9 kg/m^2^)]. There was a borderline correlation between age (*r* = 0.294), parity (*r* = 0.139) and BMI. No significant difference in educational level was found between the women in the different parity groups. A significantly higher number of women with para >5 were housewives (see Table 1). While no significant difference in median (IQR) systolic blood pressure was found, diastolic blood pressure was significantly higher in the women with para 4 or 5 [85.0 mmHg (80.0‒95.0 mmHg); see Table 1].

**Table 1:** Comparison of the factors between the parity groups in women in Sudan, 2022.

Characteristics	** **	Total (number = 408)	Nulliparity (number = 158)	Para 1–3 (number = 95)	Para 4 and 5 (number = 73)	Para > 5 (number = 82)	*P*
Age		45.0 (33.0‒55.7)	41.0 (26.0‒55.0)	40.0 (32.0‒50.0)	50.0 (35.0‒57.0)	53.0 (42.0‒60.0)	<0.001
Body mass index, kg/m^2^		26.4 (22.5‒30.5)	24.5 (19.9‒28.6)	27.1 (23.5‒30.8)	27.6 (23.7‒32.9)	27.0 (23.7‒31.3)	<0.001
Systolic blood pressure, mmHg		128.6 (119.0‒140.0)	126.5 (119.7‒135.0)	124.0 (115.0‒140.0)	128.6 (120.0‒140.0)	130.0 (118.0‒146.2)	0.160
Diastolic blood pressure, mmHg		85.0 (80.0‒92.7)	82.5 (75.8‒90.0)	85.0 (80‒90.0)	85.0 (80.0‒95.0)	81.7 (90‒103.1)	<0.001
Education level	≥Secondary	276 (67.6)	106 (67.1)	60 (63.2)	51 (69.9)	59 (72.0)	0.625
	<Secondary	132 (32.4)	52 (32.9)	35 (36.8)	22 (30.1)	23 (28.0)	** **
Occupation	Housewives	223 (54.7)	70 (44.3)	57 (60.0)	44 (60.3)	52 (63.4)	0.010
	Employed	185 (45.7)	88 (55.7)	38 (40.0)	29 (39.7)	30 (36.6)	
Hypertension	No	180 (44.1)	89 (56.3)	42 (44.2)	28 (38.4)	21 (25.6)	
** **	Yes	228 (55.9)	69(43.7)	53(55.8)	45(61.6)	61(74.9)	<0.001

In the multiple linear regression analysis, parity was not associated with systolic blood pressure, however there was a significant association between parity and diastolic blood pressure (coefficient, 0.60; *P* = 0.011; see Table 2).

**Table 2:** Multiple linear regression analysis of the adjusted factors associated with systolic and diastolic blood pressure among women in Sudan, 2022.

		Systolic blood pressure		Diastolic blood pressure	
		Coefficient (standard error)	*P*	Coefficient (standard error)	*P*
Age, years		0.34 (0.05)	<0.001	0.14 (0.04)	0.001
Parity		0.39 (0.32)	0.222	0.60 (0.23)	0.011
Body mass index, kg/m^2^		0.133 (0.14)	0.359	0.40 (0.10)	<0.001
Family history of hypertension	No	Reference		Reference	0.001
	Yes	2.38 (1.78)	0.182	2.01 (1.35‒2.99)	
Para	Nulli	Reference		Reference	
	1‒3	0.20 (2.36)	0.932	0.75 (1.71)	0.660
	4 and 5	−0.97 (2.59)	0.707	1.57 (1.87)	0.402
	>5	3.04 (2.53)	0.229	6.61 (1.82)	<0.001

**Figure 1:**
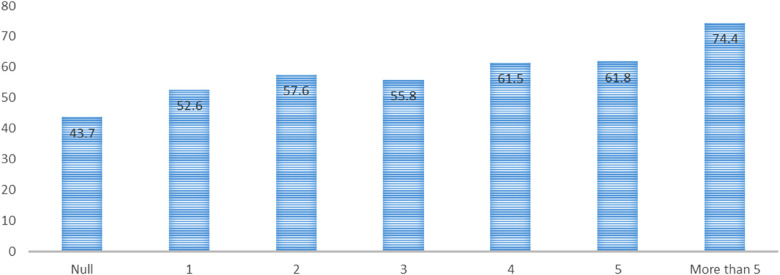
The prevalence of hypertension by parity among Sudanese women in 2022.

A total of 228 women (55.9%) had hypertension, 71 women (17.4%) had known hypertension, and 157 women (38.5%) had newly discovered hypertension. The prevalence of hypertension increased with parity and ranged from 43.7% to 74.9%. Women who had para >5 had the highest prevalence of hypertension (74.9%; see Table 1 and Figure 1). In the univariate analysis, increasing age, increasing parity, family history of hypertension, and increasing BMI were associated with hypertension. Educational level and occupation were not associated with hypertension (see Table 3). When these variables were adjusted in the multivariate analysis, increasing age (AOR, 1.03; 95% CI, 1.02‒1.05), increasing parity (AOR, 1.09; 95% CI, 1.01‒1.19), family history of hypertension (AOR, 1.79; 95% CI, 1.15‒2.77), and increasing BMI (AOR, 1.09; 95% CI, 1.05‒1.13) were associated with hypertension. Next, we removed parity as a continuous variable and entered the parity groups into the model. In this case, compared with the nulliparity (reference), para 1 to 3 (AOR, 1.35; 95% CI, 0.77‒2.38), para 4 or 5 (AOR, 1.37; 95% CI, 0.73‒2.55) was not associated with hypertension. Women with para >5 (AOR, 2.40; 95% CI, 1.26‒4.58) were at higher risk of hypertension (see Table 4).

**Table 3:** Univariate analysis of the factors (unadjusted) associated with hypertension among women in Sudan, 2022.

		Women with hypertension (number = 228)	Women without hypertension (number = 180)	OR (95% CI)	*P*
		Median (interquartile range)			
Age, years		50.0 (38.0‒60.0)	38.0 (28.0‒50.0)	1.04 (1.02‒1.05)	<0.001
Para		3 (0‒6)	1 (0‒4)	1.17 (1.09‒1.26)	<0.001
Body mass index, kg/m^2^		27.6 (24.0‒31.3)	24.3 (19.5‒28.3)	1.10 (1.06‒1.14)	<0.001
		Frequency (proportion)	** **		
Education level	≥Secondary	154 (67.5)	122 (67.8)	Reference	0.960
	<Secondary	74 (32.5)	58 (32.2)	1.01 (0.66‒1.53)	
Occupation	Housewives	132 (57.9)	91 (50.6)	Reference	0.140
	Employed	96 (42.1)	89 (49.4)	1.34 (0.90‒1.99)	
Family history of hypertension	No	100 (43.9)	110 (61.1)	Reference	0.001
** **	Yes	128 (56.1)	70 (38.9)	2.01 (1.35‒2.99)	
Para	Null	69 (30.3)	89 (49.4)	Reference	
	1‒3	53 (23.2)	42 (23.3)	1.62 (0.97‒2.71)	0.063
	4 and 5	45 (19.7)	28 (15.6)	2.07 (1.17‒3.65)	0.012
	More than 5	61 (26.8)	21 (11.7)	3.74 (2.08‒6.74)	<0.001

**Table 4:** Multivariate analysis of the adjusted factors associated with hypertension among women in Sudan, 2022.

		All women (408)		Women with age ≥ 50 years		Women with age <50 years	
		OR (95% CI)	*P*	OR (95% CI)	*P*	OR (95% CI)	*P*
Age, years		1.03 (1.02‒1.05)	<0.001	‒		‒	
Parity		1.09 (1.01‒1.19)	0.025	1.15 (1.2 ‒1.29)	0.018	1.07 (0.95‒1.20)	0.242
Body mass index		1.09 (1.05‒1.13)	<0.001	1.05 (0.99‒1.11)	0.091	1.13 (1.07‒1.19)	<0.001
Family history of hypertension	No	Reference	0.009	Reference		Reference	0.156
	Yes	1.79 (1.15‒2.77)		2.73 (1.34‒5.53)	0.005	1.49 (0.85‒2.59)	
Para	Nulli	Reference		Reference		Reference	
	1‒3	1.35 (0.77‒2.38)	0.287	1.31 (0.48‒3.55)	0.592	1.41 (0.72‒2.77)	0.313
	4 and 5	1.37 (0.73‒2.55)	0.321	2.33 (0. 84‒6.45)	0.103	1.02 (0.44‒2.36)	0950
	>5	2.40 (1.26‒4.58)	<0.001	2.73 (1.11‒6.73)	0.028	2.44 (0.99‒5.99)	0.052

We then divided the women into two age groups (≥50 and <50 years). In the women aged ≥50 years, increasing parity was associated with hypertension (AOR, 1.15; 95% CI, 1.2‒1.29). Compared with the nulliparous women (reference), para 1 to 3 (AOR, 1.31; 95% CI, 0.48‒3.55) and para 4 or 5 (AOR, 2.33; 95% CI, 0.84‒6.45) were not associated with hypertension. Para >5 (AOR, 2.73; 95% CI, 1.11‒6.73) was associated with hypertension (see Table 4). In the women aged <50 years, parity and parity groups were not associated with hypertension (see Table 4).

## 4. Discussion

The main findings of this study were a higher hypertension prevalence rate, and after adjusting for age and BMI, parity and increasing parity became significant risk factors for developing hypertension among Sudanese women. The hypertension prevalence rate among the Sudanese women (55.9%) in our study was comparatively higher than that obtained in eastern Sudan (40.8%) (19) and in some African countries such as Ethiopia (19.1%) (29) and Ghana (16%) (11). The differences in hypertension prevalence rate could be explained by the differences in sodium intake, potassium intake, alcohol consumption, obesity, nutrition, and physical activity across the regions (1, 2). The main findings of this study indicate that after adjusting for age and BMI, increasing parity was associated with hypertension (in terms of diastolic blood pressure) and women aged ≥50 years. Parity was not associated with systolic blood pressure or hypertension in the women aged <50 years.

The present study indicates that compared with nulliparity (reference), para 1 to 3 and para 4 or 5 are not at higher risk of hypertension. Para > 5 are 2.40 times more likely to have hypertension. This is in concordance with the results obtained in Mali, in which women with para ≥5 had significantly higher blood pressures (in terms of increased systolic blood pressure) than those with para 1 to 3 (30). In Ghana, women (ages 45–49 years) with para 2 or 3 had a higher likelihood of being hypertensive than younger and nulliparous women (11). A similar finding was observed in our study among Chinese post-menopausal women with para ≥5 had higher blood pressures than women with para 0 or 1; however, parity was not associated with hypertension in pre-menopausal women (15). Similarly, Turkish women with para ≥4 were at a higher risk of having hypertension than those who had para less than 4 (31). Likewise, in rural Bangladesh, high parity was positively associated with a risk of hypertension among women with obesity who had ≥4 pregnancies compared with those aged 15–75 years who only had one pregnancy (32). A previous study showed that in Iran, compared with para 2, para ≥3 was significantly associated with hypertension; however, these findings were mainly among younger women, that is, <50 years of age (12).

By comparison, Khalid (2006) reported no association between parity and hypertension among 441 women aged between 15 and 60 years in Abha, Saudi Arabia (16). A Swiss study recruited 2,837 women aged 30–73 years and demonstrated that parity had a significant adverse effect on the development of hypertension in women at 60 years; however, parity had a protective effect against hypertension in women aged <40 years (9). Giubertoni et al. reported that in Italy, while parity is associated with early hypertension during the transition to menopause, parity is not associated with hypertension in the post-menopausal period (13). In Bangladesh, women with para 1 as the reference, diastolic blood pressure was higher in nulliparous women and in para ≥3. The association between increased diastolic blood pressure and nulliparity was mainly observed in women aged >45 years, and the same association was observed among women in Bangladesh (8). Likewise, a global epidemiological study in sub-Saharan Africa demonstrated higher diastolic blood pressures (6). Our study showed that a family history of hypertension was not associated with hypertension among women with increased parity, which may reflect the heterogeneity of essential hypertension.

The results of these studies must be compared with caution. The discrepancies and modelling differences in the methods used in these studies were obvious: some studies compared women with high parity (≥5 children) with those with low-to-moderate parity, and some studies did not include nulliparous women in their analyses. Differences in lifestyle, cultural factors, genetic influence, and hypertension prevalence rate might have contributed to the differences in the results of these studies and hypertension prevalence rates among different populations (33, 34).

Parity exposes women to the risk of clinical placental syndrome (pregnancy loss, foetal growth restriction, and pre-eclampsia) as a result of altered uterine and intervillous blood flow, which is linked to inflammatory processes that lead to maternal vascular endothelial dysfunction and permanent vascular damage, thereby accelerating the development of atherosclerosis, hypertension, and cardiovascular diseases (35). Higher parity has been associated with increases in some inflammatory markers (fibrinogen, D-dimer, GlycA, high-sensitivity C-reactive protein, and interleukin-6 levels), which reflect increased risks of cardiovascular diseases and metabolic syndrome (36). In addition, the loss of the protective effect of oestrogen in postmenopausal women might lead to endothelial dysfunction and increased BMI, which are the main negative indicators of hypertension, particularly among women aged >50 years (37). The renin-angiotensin-aldosterone system in females is influenced significantly (38). Our study and several previous studies have documented significant associations between parity, BMI, and hypertension (4, 24, 28, 32). The prevalence 28.2% of obesity in the current study was slightly lower than the prevalence (33.5%) of obesity reported in eastern Sudan (19).

Several studies have reported a significant association between increasing parity and metabolic syndrome (obesity, diabetes mellitus, and dyslipidaemia), which is associated with oxidative stress and inflammation that induces endothelial dysfunction, vascular stiffening, atherosclerosis, and hypertension (39, 40). Furthermore, the physiologic cardiometabolic changes associated with pregnancy, such as insulin resistance, increased plasma glucose, weight gain, dyslipidaemia, and cardiovascular complications, increase the potential risk for developing hypertension (12, 41, 42). However, the previous studies have found positive correlations in women with a much lower number of children than what the current study is reporting. Perhaps, some other possible contributors to hypertension such as geographic location and high levels of stress among these women who raised 5 or more children and were homemakers.

## 5. Conclusion

The hypertension prevalence rate in the Sudanese women in this study was significantly high, and that parity at 5 or more is linked to hypertension.

## 6. Limitation

This study has certain limitations that should be considered. The study was a questionnaire-based survey conducted over a 3-month period. The participants' reproductive histories were self-reported, which might have increased the possibility of misclassification of parity and gravidity, particularly among the older women. Similarly, the self-reporting of menopausal status might have resulted in some misclassification. In addition, other risk factors such as history of gestational hypertension or preeclampsia, salt intake, physical exercise, oral contraceptive use, smoking, alcohol consumption, lipid profile, and blood sugar status were not assessed. Moreover, all the data obtained (apart from the blood pressure measurements) were declarative, so descriptive elements regarding the causes of secondary hypertension and other factors were lacking.

## Data Availability

The original contributions presented in the study are included in the article/Supplementary Material, further inquiries can be directed to the corresponding author.
